# Subjective health of undocumented migrants in Germany – a mixed methods approach

**DOI:** 10.1186/s12889-015-2268-2

**Published:** 2015-09-19

**Authors:** Anna Kuehne, Susann Huschke, Monika Bullinger

**Affiliations:** Department of Medical Psychology, Universitaetsklinikum Hamburg-Eppendorf (UKE), Martinistr. 52, 20246 Hamburg, Germany; School of Public Health (SPH) & African Centre for Migration and Society (ACMS), University of the Witwatersrand, 27 St Andrews Road, Parktown, 2193 South Africa

**Keywords:** Undocumented migrants, Illegal migrants, Migration, Immigrants, Subjective health, Health-related quality of life, Health status, Access to care, Social determinants of health, Germany

## Abstract

**Background:**

Health of migrants is known to be above-average in the beginning of the migration trajectory. At the same time reports from non-government organisations (NGOs) suggest that undocumented migrants in Germany tend to present late and in poor health at healthcare facilities. In this paper, we explore the health status of undocumented migrants with a mixed method approach including complementary qualitative and quantitative datasets.

**Methods:**

Undocumented migrants attending a NGO based in Hamburg, Germany, were asked to fill in the SF-12v2, a standardized questionnaire measuring health-related quality of life (HRQOL). The SF-12v2 was analyzed in comparison to the U.S. American norm sample and a representative German sample. Differences in mean scores for HRQOL were evaluated with a *t*-test and with a generalized linear model analyzing the impact of living without legal status on HRQOL. The quantitative research was complemented by a qualitative ethnographic study on undocumented migration and health in Berlin, Germany. The study included semi-structured interviews, informal conversations and participant observation with Latin American migrants over the course of three years. The study focused on subjective experiences of illness and health and the impact of illegality on migrants’ health and access to health care.

**Results:**

HRQOL was significantly worse in the sample of undocumented migrants (*n* = 96) as compared to the U.S. American sample (*p* < 0.005). Living without legal status displayed a significant negative effect on subjective mental and physical health (*p* ≤ 0.003) in the generalized linear model when adjusted for age and gender compared to the representative German population sample. The ethnographic study, which included 35 migrants, identified socio-economic conditions, the subjective experiences of criminalization, and late presentation at healthcare-facilities as the three main factors impacting on health from migrant perspective.

**Discussion:**

The present research suggests a high morbidity and mortality in this comparatively young population. The ethnographic research confirms negative impacts on health of social determinants in general and stressassociated with living without legal status in particular, both are further aggravated by exclusion from health care services. In addition to the provision of health care it appears to be important to structurally tackle the underlying social conditions which affect undocumented migrants’ health.

**Conclusions:**

Living without legal status has a negative impact on health and well-being. Limited access to care may further exacerbate physical and mental illness. Possibilities to claim basic rights and protection as well as access to care without legal status appear to be important measures to improve health and well-being.

## Background

The number of undocumented migrants in Germany is estimated to range from 100,000 up to 1,000,000 [1, 2]. While the population of undocumented migrants is diverse in reasons for migration as well as in terms of duration and nature of stay in Germany, the majority is reported to be young adults between 20 and 40 years of age [2, 3]. Although undocumented migrants are a young and healthy population at the beginning of their migration history [4, 5], they are exposed to negative social determinants of health on a large scale [6–8]. Poor living and working conditions impact on health; at the same time access to health care is complicated [3, 6–8]. Data on health status of undocumented migrants, which can inform adequate health care planning is scarce [1, 8]. In the absence of objective indices that are commonly used to measure health and provide information on the need for health care provision, health-related quality of life (HRQOL), and self-reported health and barriers to healthcare of undocumented migrants offer unique insights into the health status and challenges of a hidden population.

### Legal situation

Migrants without a valid residency permit in Germany are referred to as undocumented migrants. Every state institution in Germany is obliged to report migrants, who cannot provide a valid residence permit, to the police or migration authorities, according to §87 of the German Residence Act [Aufenthaltsgesetz] with few exceptions. As a result contact to official institutions for any purpose including claiming basic human rights, such as labor rights, protection from crime or access to health care can result in deportation and makes undocumented migrants vulnerable to exploitation.

Access to medicine for undocumented migrants is regulated by §§4 and 6 of the German Asylum Law [Asylberwerberleistungsgesetz]. It offers access to health care for acute or painful diseases and obstetric conditions. In practice accessing health care according to the German Asylum Law requires sharing personal data of undocumented migrants with state institutions in accordance with §87 German Residence Act, which can result in deportation when seeking health care.

### Coping with disease and access to health care

Since access to health care under the German Asylum Law is effectively associated with a risk of deportation for undocumented migrants, other ways of dealing with health conditions are usually employed. Presence of disease is neglected as long as possible and health services are only accessed if self-medication and informal network support proved unsuccessful [3, 6–12]. When informal networks fail, paying privately for treatment or seeking support from a non-government-organization (NGOs) are alternative options to access care. ‘Medibueros’ and ‘Medinetze’ are local NGOs available in 33 German cities providing access to health care for migrants, anonymous and free of charge within a network of health care workers and hospitals. Availability of treatment remains incomplete and dependent on donations [13, 14].

### Health status

Undocumented migrants have been reported to be younger and less healthy than common patients at health facilities [15, 16]. Furthermore according to NGOs their health problems are more severe and further advanced when accessing care [15, 17]. Living and working conditions are known to be important determinants influencing health and well-being and undocumented migrants are more likely than the general population to experience difficult living and working environments and have less access to care [3, 8, 18, 19]. Difficulties in claiming basic rights and constant fear may aggravate existing health problems [7, 8, 20].

### Subjective health of undocumented migrants

Since undocumented migrants systematically elude themselves from empirical research, representative data on the health status of undocumented migrants is unavailable. While undocumented migrants do not appear in official registration data nor in hospital and health insurance records, self-reported health-related quality of life (HRQOL) can provide insights into the health and well-being of this hidden population.

Health-related quality of life (HRQOL) represents a broad concept of health. It is a multidimensional construct incorporating physical and mental well-being as much as social relations and functional competences [21]. HRQOL has been shown to not only correlate significantly with morbidity and mortality, but also to be a valid indicator to predict them [22–25]. Furthermore the need of outpatient and inpatient treatment and future health care needs are predictable from HRQOL [26]. Self-rated HRQOL measurements can offer insight into the current and future health status of undocumented migrants.

A European review found migrants to rate their health poorer than the usual population [27], some evidence was found that not only migration status but also socioeconomic factors, discrimination, language barriers and social support influenced self-rated health [27, 28]. For legal migrants in Germany a negative association of perceived discrimination and HRQOL was established [29]. While HRQOL of undocumented migrants has not been studied so far with a standardized instrument, very few investigations on subjective health of undocumented migrants – using a single-item question to assess self-rated health - have been conducted [30–33]. Consistently undocumented migrants rated their health worse than legal migrants or the local population [30–33]. One investigation found fear of deportation to be independently associated with low self-rated health in regression analysis [30]. Accordingly more than 60 % of 102 undocumented migrants surveyed by Médecins Sans Frontières in Sweden reported deterioration of mental and physical health during their stay without a valid residence permit [6]. Very few ethnographic studies focusing on health of undocumented migrants have been carried out so far – all of them indicated a negative impact of living without legal status on health [8, 20].

Since data on health of undocumented migrants is scarce, the objective of the present study is to assess the health status of undocumented migrants by using a standardized HRQOL-questionnaire combined with qualitative ethnographic analysis adding insight into perspectives on health of undocumented migrants.

## Methods

We applied a mixed methods approach to combine the advantages of a standardized instrument to measure subjective health with the benefits of qualitative research in generating knowledge about experiences, biographies and social interactions. Triangulation of different types of data and research methods increases the validity of data and allows gaining more comprehensive insight into health of undocumented migrants.

### Ethics

Undocumented migrants are frequently confronted with situations that may appear threatening to them, given their inability to claim their rights and the possibility of being denounced to authorities by others. An utmost concern in designing the study was to balance gathering comprehensive information against the danger of keeping migrants from participating due to their sensitivity to data collection. Furthermore much effort was made to ensure participants in both studies understand the voluntary and anonymous nature of the research. The quantitative study was approved by the ethical committee of the Medical Association of Hamburg, Germany (Aerztekammer Hamburg). For ethnographic studies in Germany, ethical review is not formalized; the qualitative study followed the ethics declaration of the German Anthropological Association (DGV e.V.). In both studies the principles of the declaration of Helsinki regarding ethics in medical research were followed. Informed consent was obtained verbally in both studies. In the quantitative study, migrants who were not capable of communicating in English, German, Spanish or French themselves or through an interpreter of their choice were not included in the study. The interviews and conversations in the qualitative ethnographic study were all conducted in Spanish. The names of all participants were changed.

### Sampling, study design and measures

#### Quantitative study

The present sample consists of migrants who visited the organization for medical counselling of refugees and migrants in Hamburg (short: Medibuero) within a research period of six months in 2007. The Medibuero is a local NGO organizing health care anonymously and free of charge for undocumented migrants.

The Medibuero was at the time of research the only NGO offering access to health care for undocumented migrants in Hamburg. All migrants visiting the Medibuero for a doctor’s appointment, who consented to participate, were asked to complete a standardized questionnaire about HRQOL after they had been attended to by the Medibuero staff. Questionnaires were filled in by the migrants on the spot in the Medibuero office or, where help was needed, researchers assisted participants in filling out questionnaires. Age and gender were recorded together with physical or mental health complaints for which medical care was sought.

We used the standardized questionnaire *Short Form-12v2 Health Survey* (SF-12v2) constructed by Ware *et al.* to measure HRQOL [34]. Among the instruments available to assess HRQOL, the *Short Form-36 Health Survey* (SF-36) and its adapted 12-item version, the SF-12v2, are reliable and valid generic HRQOL measurement instruments widely used in international research [34–37]. In addition the SF-12v2 shows good correlation between negative self-rated health and the effect of negative social determinants. It offers the possibility to measure the perceived functional and emotional health status of populations and their affectedness by risk factors [38]. It was validated in all languages relevant to the current study: Bulgarian, French, English, German, Hungarian, Latvian, Lithuanian, Polish, Portuguese, Rumanian, Russian, Spanish and Turkish. We used the 12-item standard version referring to perceived health within the past 4 weeks. It measures eight dimensions of subjective health: ‘physical functioning’ (PF), ‘role limitations due to physical problems’ (RP), ‘bodily pain’ (BP), ‘general health’ (GH), ‘vitality’ (VT), ‘social functioning’ (SF), ‘role limitations due to emotional problems’ (RE) and ‘mental health’ (MH) [34]. All eight dimensions can be scored separately and, in addition, can be used to calculate two summary measures: The Physical component summary (PCS) and the mental component summary (MCS) [34]. Summary measures were only computed if all items were filled in completely.

#### Qualitative study

Sampling included individual first contacts through a community center, NGOs and friends complemented by snowball-sampling. Interviews were carried out between May 2008 and April 2011. Additional follow-up interviews were conducted in person and via email on several occasions in 2012 and 2013.

The case examples presented in this paper were purposefully selected to exemplify the diversity of the sample, and to highlight specific issues related to subjective experiences of health. The conclusions drawn from these cases are, however, based on the qualitative analysis of the entire data set.

Methods included semi-structured interviews, addressing the participant’s biography, migration history, living and working conditions, and illness experiences. Notes based on informal conversations provided additional information. Furthermore, two focus-group discussions were carried out in which the research participants discussed definitions of health and sickness, and different forms of healing, including biomedicine, spiritual healing, and medicinal plants. The conversations and semi-structured interviews were complemented by participant-observation. The researcher spent several hundred hours with undocumented Latin Americans at their homes, their workplaces, or at church, and accompanied them to appointments with doctors and lawyers. Extensive field notes were taken during and after these encounters.

### Data analysis

#### Quantitative study

Data analysis was conducted with SPSS 15.0 and in accordance with the instructions from the SF-12v2 manual [34]. The results of the Sf-12v2 are scored based on U.S. American norms [34]. For norm-based scoring the results of the norm sample in the eight dimensions are assigned a mean value of 50 and a standard deviation of 10. With norm based scoring, results in any given sample are analyzed in relation to the norm sample – values above 50 indicating better health and values below 50 indicating worse health than the norm sample [34]. Utilizing U.S. American norm values is recommended for non-U.S. populations to ensure comparability of the data [37].

Initially the eight dimensions of HRQOL are descriptively displayed in their relation to the norm values. Comparison with the norm values was performed with a *t*-test for difference in means. Further analysis of the eight dimensions was not possible as data of American and German comparison groups, who suffer from health complaints, was only available for the summary measures of subjective mental and physical health but not for the eight dimensions of HRQOL.

Thereafter the summary measures of subjective mental and physical health, MCS and PCS, were compared to the U.S. American norm sample, to allow for international comparison, and to a representative German sample, to account for the local context. Both samples consist of individuals, who suffer from health complaints, to ensure comparability with the obtained sample that consulted the Medibuero to access healthcare.

Comparison with the subgroup of the U.S. American norm sample that indicated presence of one or more health conditions was performed with a *t*-test for difference in means. Values of the U.S. norm sample are published for the general population and the subgroup of cases that indicated having a physical complaint. The general population has a mean age of 50.7 years and 59.6 % are women [34]. No such information or further granularity of data is available for the subgroup with physical complaints.

German population norms for the SF-12v2 are unavailable. However in a representative German survey conducted by the Robert KochInstitute (RKI) in 1998, the German National Health Interview and Examination Survey 1998 (BGS98), the SF-12v1 was used to measure HRQOL on population basis [39, 40]. For the BGS98 a representative German sample was selected and 6,964 participants were interviewed with the SF-12v1 [41]. Differences in response were adjusted for by weighting factors developed by the RKI to ensure representativeness for the 18 to 79 year old German population [41].

Summary scales for mental and physical health that were generated using SF-12v2 are comparable to SF-12v1 summary scales when scores are based on the same norms and multiplication-factors for adjustment from version 1 to version 2 are utilized. For comparison we used original SF-12v1 data derived from the BGS98 and re-scored and adjusted it with QualityMetric Health Outcomes™Scoring Software 4.0. Comparison was performed against the subgroup of the BGS98-sample that reported having visited a physician within the past 4 weeks for an acute or chronic health problem. The influence of lack of legal status, age and gender was analyzed using a generalized linear model with robust estimators.

#### Qualitative study

The qualitative data was analyzed following the grounded theory approach [42], using Atlas.ti software. The interview transcripts and field notes were coded parallel to fieldwork, and analytical categories and conclusions were developed inductively over the course of the research project. Two forms of coding were employed: *in-vivo* coding, which generated analytical categories based on the actual words used by research participants, and open coding, were codes were generated by the researcher based on a close reading of the textual data. Codes were complemented by *memos*, i.e., written explanations of codes or comments connecting various codes and insights. Interview data, conversational notes and field notes were triangulated. Emerging categories and analytical insights were discussed with research participants to further ensure data validity.

## Results

### Quantitative study

#### Sample description

96 of 147 migrants who accessed the Medibuero personally- during the timeframe of six months - were included in the study (65.3 %). Of the 51 migrants not included in the study 17 were excluded for language barriers, 16 did not want to participate, nine had no time to participate, seven needed immediate medical treatment not allowing study participation and two refused to participate because of fear.

58.3 % originated from Latin America or the Caribbean, 17.7 % were from Africa and 16.7 % came from Southeast- or East-Europe or countries formerly belonging to the Soviet Union and 7.3 % came from other regions. 98.6 % of the migrants reported to seek medical advice for a physical complaint, 1.4 % came because of a mental health problem. Sociodemographic characteristics of the sample and the comparison groups are displayed in Table 1.

**Table 1:** Sociodemographic characteristics of the sample of undocumented migrants for the quantitative study and the different comparison groups

Sample and comparison groups	n	Mean age in years	% female	Statistical comparison performed
Quantitative study sample of undocumented migrants	96	37	72 %	-
U.S. American norm sample, complete sample [34]	7069	51	60 %	*T*-test of differences in means of 8 dimensions of health, see Fig. 1.
U.S. American norm sample, subgroup of participants indicating one or more physical disease [34]	2329	n. a.	n. a.	*T*-test of differences in means of subjective physical and mental health summary scores, see text.
Representative German sample, subgroup of participants indicating having visited a physician for an acute or chronic disease in the past four weeks [39]	1955	51	56 %	*T*-test of differences in means of subjective physical and mental health summary score by age-group and gender, see Table 2.
				Generalized linear regression model, see Table 3.

*n. a.* = not available

#### Eight dimensions of HRQOL of undocumented migrants

**Fig. 1:**
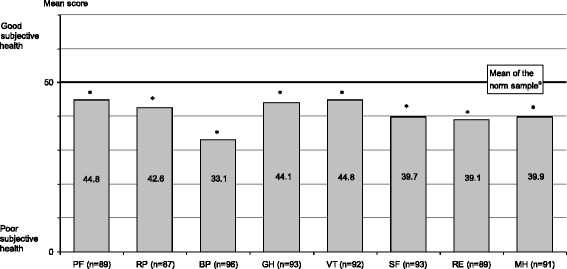
Mean scores of undocumented migrants in the eight dimensions of HRQOL measured with SF-12v2 compared with U.S. American norm. ^a^ U.S. American norm sample [34] PF=‘physical functioning’. RP = ‘role limitations due to physical problems’ BP = ‘bodily pain’ GH = ‘general health’ VT = ‘vitality’ SF = ‘social functioning’ RE = ‘role limitations due to emotional problems’ MH = ‘mental health’. * Difference in means significant at p < 0.001

Mean scores in all eight dimensions of HRQOL in the sample of undocumented migrants were significantly lower than the U.S. American norm scores. Mean scores more than a standard deviation below the U.S. American norm were observed in the dimensions of ‘role limitations due to emotional problems’, ‘social functioning’, ‘mental health’ and ‘bodily pain’, see Fig. 1.

#### Summary scores for mental and physical HRQOL of undocumented migrants

Summary scores could be computed for 79 undocumented migrants. Mental and physical summary scores health showed significant lower scores in undocumented migrants when compared to representative U.S. American and German samples of people, who suffered from illnesses as well.

Undocumented migrants seeking health care at the Medibuero scored a mean of 43.0 points in the subjective physical health summary measure and 40.5 points in the subjective mental health summary measure. Both scores were significantly (*p* < 0.001) lower than the scores of the sample of U.S. Americans with at least one health condition (47.7 points and 51.7 points, respectively) [34].

Scores for mental and physical health indicated worse HRQL in undocumented migrants when compared to the subgroup of the representative German sample that visited a physician within the past 4 weeks for an acute or chronic disease, see Table 1. Differences proved to be significant in the generalized linear model with robust estimates, see Table 2.

**Table 2:** Comparison of HRQOL measured with SF-12v2 of undocumented migrants with a German sample

Summary Scales	Subgroups	Undocumented migrants				German sample^a^			
		N	M	SD	Mdn	N	M	SD	Mdn
Subjective Physical Health	20–34 years	31	43.98	5.10	43.63	391	47.68	8.59	49.66
	35–49 years	30	43.82	6.49	43.26	431	44.77	10.10	47.23
	50–64 years	12	39.05	6.03	40.47	590	41.12	9.95	42.37
	Men	23	43.76	7.02	43.28	865	43.90	10.01	45.95
	Women	56	42.61	5.36	42.85	1090	42.39	10.45	43.73
	Total	79	42.95	5.87	43.07	1955	43.06	10.28	44.79
Subjective Mental Health	20–34 years	31	39.87	7.75	38.79	391	50.65	8.49	52.65
	35–49 years	30	40.58	9.28	41.28	431	50.49	9.59	53.02
	50–64 years	12	40.58	10.49	39.74	590	50.92	10.21	53.96
	Men	23	39.67	7.96	38.79	865	52.38	9.01	55.04
	Women	56	40.88	8.82	41.28	1090	50.16	9.84	52.62
	Total	79	40.52	8.55	40.19	1955	51.14	9.55	53.57

*M* Mean, *SD* Standard Deviation, *Mdn* Median. ^a^The German sample constitutes a subgroups of the representative German sample surveyed with the BGS98. The subgroup includes all participants that visited a physician for an acute or chronic complaint within the past 4 weeks

**Table 3:** Generalized linear model with robust estimates for the effect of gender, age and migration status on HRQOL measured with SF-12v2

	Subjective Physical Health			Subjective Mental Health		
	Estimate	SD	*p*-value	Estimate	SD	*p*-value
Constant	51.94	.70	<= .001	47.66	.67	<= .001
Men	1.21	.44	.006	2.27	.42	<= .001
Women	Reference	.	.	Reference	.	.
Undocumented migrants	−2.22	.75	.003	−9.63	1.05	<= .001
German sample^a^	-	Reference	.	-	.	.
Age	-.19	.01	<= .001	.05	.01	<= .001

Dependend variable: Subjective physical health, Subjective mental health

*SD* Standard deviation

^a^The German sample constitutes a subgroups of the representative German sample surveyed with the BGS98. The subgroup includes all participants that visited a physician for an acute or chronic complaint within the past 4 weeks

Generalized linear model with robust estimates indicates a significant negative effect of living without legal status on mental and physical health. Mental health summary scores of undocumented migrants were reduced by 20.2 %, physical health summary score by 4.3 % in comparison to the subgroup of the representative German sample with reported health care consumption, see "Reference" Table 3.

### Qualitative study

#### Sample description

A total of 35 migrants from various Latin American countries took part in the qualitative ethnographic study. The research participants were either undocumented at study entry, or shared their experiences of having lived in Germany illegally in the past. The majority of the research participants in the study were women between 20 and 45 years of age, many of them mothers. All of the research participants had completed secondary education, and many had received vocational training or studied at a university.

#### Key results of qualitative analysis

The analysis of interview transcripts and field notes showed that prevalent stressors are precarious socioeconomic conditions and a constant fear of denunciation, detention and deportation. All research participants had come to Germany to either work or study in order to provide a better life for themselves and, in most cases, also for dependent family members. Many of the participants experienced phases of homelessness, unemployment or poverty, usually at the beginning of their stay in Germany. Over the course of time these conditions usually stabilized: the majority of the research participants found paid work, usually as cleaners and child-minders or in the service industry, and lived in their own apartment. However the absence of employment contracts or rental agreements, and the constant threat of losing work and housing resulted for most study participants in perceived instability of daily routine. This precarious situation is exacerbated by the fact that many undocumented migrants have debts due to the costs of migration, and their income in Germany often lies significantly below what they had hoped for and counted on. Getting sick or not being able to work due to childcare responsibilities, or even worse, being arrested and subsequently deported would have significant negative economic consequences for migrants and for their families, in addition to the emotional trauma caused by imprisonment and an involuntary return to the country of origin.

The majority of participants reported that the perceived threat of deportation was impacting on daily life as much as on accessing health care as consulting a hospital or a physician was considered a potential way of being identified and subsequently transferred to the country of origin. All study participants experienced problems in accessing health care and many reported deterioration of health status due to the lack of access. For undocumented migrants, who had either just recently moved to Germany, and/or had not gathered the necessary knowledge yet to know where and how to access the non-governmental healthcare providers, the ethnographic study provides evidence that the common response to physical complaints was usually to “bear” the pain or self-medicate.

The following case studies exemplify precarious socioeconomic conditions experienced by undocumented migrants and the effect of criminalization on physical and emotional well-being of undocumented migrants.

#### Case example 1

Dominga came from La Paz, Bolivia to Berlin in 2009. The woman in her early twenties was invited over on a tourist visa by a Bolivian family who employed her (illegally) as a care-taker for their children. They had paid for her travel costs, and expected her to pay off her debt by what came down to working for less than one Euro per hour. Half a year after she arrived, when her visa had already expired, Dominga realized that she was pregnant. After she had digested the shock of the unplanned pregnancy, Dominga decided to keep the child, and to make a life for herself and her son in Berlin. The following years were shaped by hardship: as an undocumented migrant and single mother, Dominga relied heavily on friends and non-governmental organizations to get by. She received perinatal and postnatal care through the Berlin-based Medibuero, one of the non-governmental organizations introduced above. The rent for the apartment she lived in was paid for by a network of women supporting undocumented Latin American migrants. A few months after her son Christian was born, Dominga started working as a cleaner in private households. She could only work for a few hours at a time, as she had no one to take care of her son while she was working. When her child was not yet a year old, she had to leave the apartment she was living in because her former employer, who felt that she had betrayed her by leaving her ‘job’ and not paying off her debt, had found out where she lived. Dominga was worried that she would denunciate her to the police. She explained in an interview:

*“So much worries! My ears, I got an infection, blood came out of my ears. From all the stress, from the shock that I got when I needed to move out of the apartment. Look, I got sick because of that. I asked Stefan [a friend who volunteered for the Medibuero]: ‘Please, please, I need to see a doctor.’ After that, I got better, but then I got really, really sick again. It was the fear of the police, that the police would come find me. (…) A friend told me I could come live with her for a while, so I moved all my stuff, imagine, doing all that while being so sick. (…) With my ears, I was so ill, I said to myself, I want to rest, I am sick. If the police come – they come, I don’t care. I’ll go back [to Bolivia]. I couldn’t sleep. It was difficult, you know. I don’t know why I am suffering…so much stress…and with my son [starts crying], I am sick, my ears hurt… Sometimes I don’t know what to do.”* (Follow-up interview, 17.10.2012)

Dominga was ill for almost one year. In addition to the ear infection, both she and her son suffered from a persistent cough and were eventually diagnosed with pneumonia by a Medibuero doctor and hospitalized for 10 days. In the end, she decided to return to Bolivia:

*“There is no security here. What happens to my son if I get sick again? Who takes care of him? It is too much stress, too much fear.”* (Informal conversation, 13.12.2012).

#### Case example 2

Jaime, a young man from Chile, had come to Europe – first to Italy and then to Germany – with the aim to study. His student visa application failed, however, and he ended up as an undocumented migrant in Berlin. The four-hour interview was not recorded, as he was concerned about confidentiality, so the following narrative is based on extensive notes. At the time of the interview, Jaime’s status had been legalized through marriage. Looking back, he described being an ‘illegal immigrant’ as a “*trauma*” and a “*shock*”: “*Nobody tells you what it is like to be illegal.*” Like most other research participants, Jaime came to Germany to improve his life, to “*get ahead*” by studying and earning money. He suffered a great deal from what he perceived to be a failure to achieve his aims. He said:

“*I was very depressed, it was a bad life. It was tearing me apart. To see your dream shattered means to see yourself shattered*.”

He started drinking heavily, started “*losing reality*”, as he puts it. Linking his physical and emotional health to his living circumstances, he commented:

“*This [living illegally] really pushes you to the limit. It’s an emotional crisis; it’s a state of un-well-being. Bad alimentation, too much work, too much stress, sleeping badly. All of this directly affects the heart.” (*Interview notes, 2.8.2009).

#### Case example 3

Tabea came to Germany in 2007, together with her German boyfriend whom she met in Bolivia. She wanted to study German in order to improve her education and subsequently, her job opportunities. Like Jaime, she was denied a student visa, and once her tourist visa ended, she was illegalized. In an interview, she described how her illegal status affected her life:

*Tabea: “The situation [being illegalized] affects me, it even affects my health! Sometimes I stumble into a depression. I can’t even travel; I always have to watch out. (…) I am always scared, I have so much fear. So far, I never ran into a control. Thank God! (…) If I didn’t believe in God, I couldn’t go on here; I wouldn’t be able to bear the situation, right? Because it’s a situation… it’s a shitty situation really.”*

*Interviewer: “And how does that affect your health?”*

*T: “I am very depressed, every time I leave the apartment I say, my God, please protect me. That nothing bad happens to me, because I don’t know what’s going to happen. Because of that, I can’t live in tranquility. So, that [sighs]… that affects my health, because I am always nervous, I always live with this fear that something could happen to me. And I get depressed because I feel trapped, I can’t do anything, everything has to stay secret, I have to lie, when somebody asks me if I have a student visa, or when I met a guy, I can’t tell him the truth because I am scared that he might think something else [that she only wants to be in a relationship to get married and be legalized]… So, I have my limits, I have met people who have lived here illegally for 10 years, and I tell them: How do you live?? Tell me, how do you live?? (…) I have been here for 7 months. I can’t do it, I can’t!! I always have this fear, I always cry, I am not doing well.”* (Interview, 6.6.2008)

## Discussion

We utilized a mixed method approach to investigate subjective health of undocumented migrants and influencing factors. We demonstrated that HRQOL of undocumented migrants is significantly lower than in comparison groups, in particular mental health is rated lower and pain intensity is rated higher. The results of the qualitative research illustrate how negative social determinants, stress and fear, and exclusion from the health care system contribute to a deterioration of the health of undocumented migrants.

### Health status of undocumented migrants

Undocumented migrants rated their health significantly worse than the general population in all eight dimensions of HRQOL measured by the SF-12v2. Scores were particularly low in the dimensions assessing pain, mental health, role limitations due to mental problems and social functioning. Subjective physical and mental health was worse for undocumented migrants than for German and U.S. American comparison groups. Given the mean age in the U.S. American norm population of more than 50 years, it is likely that undocumented migrants were not only less healthy but as well much younger than their U.S. American comparison group in accordance with what has been described in the literature [15, 16]. The lower scores in physical health and higher scores in pain intensity can be due to differences in the time since having visited a physician (up to 4 weeks in the comparison group but just at the time of investigation for the undocumented migrants). However, these differences can as well represent differences in pain intensity tolerated until medical care is sought. Even though all but one undocumented migrant declared to have come to the Medibuero for a physical health problem, their mental health summary scores differed significantly from the comparison groups. The differences are possibly a result of mental health problems of undocumented that are expressed as non-specific physical health complaints, which is described in the literature as a common consultation pattern of undocumented migrants [8, 15].

The ethnographic study supports these findings, sheds light on the prevalent stressors and explains how and why the health status of undocumented migrants is often significantly worse than the health status of legal residents, as discussed below.

### Effect of socioeconomic conditions

SF-12v2 physical and mental health summary scores as well as the eight subscales correlate with social determinants of health such as socioeconomic background, education attainment, income and employment [38]. They are as such possible indicators for negative effects of socioeconomic conditions associated with life without legal status which could not be controlled for in the present study. Significantly lower scores compared to Germans for physical and mental health are likely to reflect differences in socioeconomic status and ability to participate in society.

The results of the qualitative study support this interpretation. In regard to undocumented Latin American migrants, however, this study also shows that negative social determinants are not limited to factors such as poor housing and precarious labor conditions, but are largely due to the inherent fragility of their life [10], due to lack of contracts, lack of insurance and lack of social benefits. Consequently, when undocumented migrants get sick or cannot work due to childcare responsibilities, they often risk their job and face economic hardships. In the case of Dominga, her health problems were exaggerated by her social circumstances as an undocumented migrant and single mother of an infant. She could not work as much when her son was born, relied on friends to help her out with paying rent, and had very little money left to buy nutritious food. From her point of view, these factors affected her physical as well as her mental health. Constant insecurity thus constitutes a defining feature of life without legal status, and can be viewed as a negative social determinant of health [8, 19].

### Effect of criminalization and fear

In addition to precarious socioeconomic conditions, for example job insecurity, which also affect others groups of society, undocumented migrants experience additional stress of criminalization and fear, as described in the case examples. Fear of deportation and discrimination have been shown to independently affect subjective health of migrants [8, 29, 30]. In the analysis of the dimensions of HRQOL measured by the SF-12v2, respondents were found to experience greater limitations than the general population in daily life due to mental problems, to feel less often calm and energetic and to experience greater health-related difficulties in their social activities. All of the case studies presented above exemplify the relationship between illegal status and mental ill-health. Tabea experienced significant role limitations due to her illegal status and the mental health problems that resulted from it. She did not feel that she could be herself, and isolated herself from other people. For Dominga, the pressure of being an undocumented single mother affected her mental health, and eventually led her to return to Bolivia despite the fact that she had not reached her migration goals. Jaime very clearly saw his legal situation, and the fear and stress associated with it, as a cause of “trauma” and mental suffering. Even years after his status was legalized, he still struggled to recover from the years of constant emotional distress and found it difficult to trust other people. Fear of denunciation, arrest and deportation shape how undocumented migrants interact with other people, and may lead them to isolate themselves in order to avoid denunciation [10]. These present results are in accordance with findings from qualitative and quantitative research reporting negative impact of fear of deportation and living illegally on health [3, 7, 8, 19, 30].

### Effect of delay in seeking health care

Negative impacts of socioeconomic hardships, insecurity and fear may be exacerbated by limited access to healthcare. The ethnographic study and the available literature suggest that problems in accessing healthcare are common among undocumented migrants and particularly affect the people who recently migrated [12, 28].

Long latency before physician consultation can lead to more severe symptoms, chronification, or to a general deterioration in overall health. Undocumented migrants experience more pain than the comparison groups. High scores of pain and poor general health can be indicators for long latency before accessing health care – a characteristic pattern of health care seeking behavior of undocumented migrants described in the literature [3, 6–8, 11, 15, 28]. Accordingly, physicians cooperating with the Medibuero reported a more detrimental health state of undocumented migrants when they seek care as compared to their usual patients [16]. These findings are supported by published studies suggesting more advanced diseases in undocumented migrants and poorer health as compared to legal residents [16–18]. Furthermore 75 % of a migrant sample visiting the same facility reported deterioration of health state during the time they did not access healthcare and 70.8 % of undocumented migrants reported they would have sought care earlier if it would have been possible without danger [16].

### Limitations

Quantitative study: Given the lack of available data about population size and characteristics and the seclusion of the population, no random sampling could be conducted. Age and gender distribution within the obtained sample were similar to the demographic characteristics of undocumented migrants described by other health care providers [8, 15], however male undocumented migrants are under-represented in the sample [2]. The sample consists of migrants that tried to access health care and can as such not reflect the health status of the whole population of undocumented migrants but of those who became sick during their stay without residence permit (and knew or got to know how to access healthcare with limited risk of deportation). Comparisons of HRQOL were therefore conducted against two samples that were as well affected by ill health and either indicated suffering from at least one physical disease (U.S. American comparison sample) or reported consulting a physician for a disease within the past four weeks (German comparison sample). Lack of granularity of the U.S. American comparison data did not allow for stratified analysis [34]. Unfortunately no comparable data about legal migrants in Germany is available.

Statistical tests applied were based on normal distribution. The scores of the obtained sample in the eight dimensions of subjective health were slightly skewed, for all but the dimension of general health and social functioning, to the left, indicating that differences might be overestimated. However, summary scores of the obtained sample of undocumented migrants were normally distributed, but the comparison groups displayed slightly left skewed scores [34]. Given the left skewed distribution of the U.S. American comparison group and the normal distribution of the obtained sample, the true difference in means that indicated lower scores for the undocumented migrants is likely to be larger than the result of the applied *t*-test indicates. Non-parametric tests could not be applied to the U.S. data for lack of detailed data. As part of sensitivity testing, the generalized linear model was recalculated on the basis of data transformed by complex logarithmic transformation [f(x) = ln(1/(1-(MCS/100))] to comply better with model requirements. Transformed data indicated the same grouping (negative effect of living without legal status, negative effect for female gender), similar differences and significances and nearly straight relations between age and mean scores. Similarity of results of the models based on original and transformed data indicates that the generalized linear model on the basis of the original data represents the interrelations sufficiently.

Qualitative study: Undocumented migrants constitute a diverse population, and differences in regard to region of origin, educational background, gender, and migration experience will shape the living conditions and consequently the health experiences in Germany. Compared to other undocumented migrant groups, Latin Americans in the present sample were relatively well-educated, and most of them did not migrate because of war, violence or hunger – their main reason to migrate to Europe was a lack of economic opportunities in their country of origin [43–45]. Thus, it is possible that other migrant groups, e.g., migrants who experienced physical or emotional trauma before or during their migration to Europe, are even more affected by the negative implications of illegalization discussed above.

## Conclusion

While being descriptive in nature, the present research sheds light on the health status and need for health care of a hidden vulnerable population often overlooked in health care planning. Subjective health and quality of life have for the first time been described in undocumented migrants in Germany using a valid and reliable instrument that allows inference from HRQOL to morbidity, health-care-needs and social determinants [22, 24, 26, 38]. The present research suggests a high morbidity and mortality in this comparatively young population waiting to access health care. Yet, further research is needed to specify health care needs and possibilities to overcome barriers to care. The ethnographic research confirms negative impacts on health of social determinants in general and stress associated with living without legal status in particular, both are further aggravated by exclusion from health care services. Fear of deportation affected health directly by causing mental stress and indirectly by complicating access to human rights and by contributing to long latency before health care was sought. Offering the opportunity to claim rights independently from residence permit can help to improve living and working conditions and as such impact on health. As HRQOL is known to be inversely correlated with future health care needs [24, 26], adequate access to health care will assist in preventing long term consequences of unattended health conditions. In addition to the provision of health care it is important to structurally and politically tackle the underlying social conditions which affect undocumented migrants’ health. Thus, the reduction of negative social determinants of health as much as timely and risk-free access to comprehensive health care are critical measures to improve health of undocumented migrants.
